# Anti-Inflammatory Effects of Resveratrol: Mechanistic Insights

**DOI:** 10.3390/ijms19061812

**Published:** 2018-06-20

**Authors:** Diego de Sá Coutinho, Maria Talita Pacheco, Rudimar Luiz Frozza, Andressa Bernardi

**Affiliations:** 1 Laboratory of Inflammation, Oswaldo Cruz Institute, Oswaldo Cruz Foundation (FIOCRUZ), Rio de Janeiro 21040-360, Brazil; diego_dsc_@hotmail.com (D.d.S.C.); talipacheco@gmail.com (M.T.P.); 2 Laboratory on Thymus Research, Oswaldo Cruz Institute, Oswaldo Cruz Foundation (FIOCRUZ), Rio de Janeiro 21040-360, Brazil

**Keywords:** resveratrol, inflammation, chronic diseases

## Abstract

Inflammation is the principal response invoked by the body to address injuries. Despite inflammation constituting a crucial component of tissue repair, it is well known that unchecked or chronic inflammation becomes deleterious, leading to progressive tissue damage. Studies over the past years focused on foods rich in polyphenols with anti-inflammatory and immunomodulatory properties, since inflammation was recognized to play a central role in several diseases. In this review, we discuss the beneficial effects of resveratrol, the most widely investigated polyphenol, on cancer and neurodegenerative, respiratory, metabolic, and cardiovascular diseases. We highlight how resveratrol, despite its unfavorable pharmacokinetics, can modulate the inflammatory pathways underlying those diseases, and we identify future opportunities for the evaluation of its clinical feasibility.

## 1. Introduction

Inflammation is a natural protective response of the body to infection or injury; this response helps to maintain tissue homeostasis under stressful conditions [1]. This complex, tightly regulated process serves as a rapid defense mechanism to contain potential pathogens, limit further tissue damage, and stimulate repair mechanisms; consequently, inflammation is crucial for human health [2]. Although inflammatory response processes depend on the precise nature of the initial stimulus and its location in the body, they all share a common mechanism, which consists of the following steps: (1) cell-surface pattern receptors recognize detrimental stimuli; (2) inflammatory pathways are activated; (3) inflammatory markers are released; (4) inflammatory cells are recruited; and (5) the target tissues are affected [3,4]. This complex set of events results in the cardinal signs of inflammation: pain, heat, redness, swelling, and eventual loss of function [5].

Inflammation can be acute and chronic [1]. Usually, the acute phase is initiated by tissue-resident cells that detect pathogens or trauma, and then send chemical signals which amplify the local response and recruit other cells [2]. Typically, the molecular and cellular events during acute inflammatory responses are efficient, leading to the restoration of tissue homeostasis, and thus, the complete resolution of inflammation [6]. However, altering or prolonging the activation of the inflammatory response, even for low-grade inflammation, can trigger the second stage, called chronic inflammation, which can cause more damage to a host than the pathogen itself [7]. Low-grade inflammation may persist throughout periods of life due to recurrent or persistent infections, and emerging evidence indicated that inflammation has a pivotal role in the pathogenesis of several chronic diseases, including metabolic, cardiovascular, pulmonary, and neurological disorders [8,9,10,11]. Moreover, studies showed an association between inflammation and some types of cancers [2,12].

Inflammation therapy is based on the use of non-steroidal anti-inflammatory drugs (NSAIDs), which possess multiple side effects and have limited efficacy. These drugs are potent inhibitors of cyclooxygenases 1 and 2 (COX-1 and COX-2). Despite COX-2 being induced by inflammation to trigger the production of pro-inflammatory prostaglandins (PGE2 and PGD2), COX-1 is the constitutive isoform involved in homeostatic processes. Because NSAIDs inhibit both enzyme isoforms, its continuous use can lead to damage to the gastrointestinal tract—the main side effect of these drugs. Therefore, the development of selective COX-2 inhibitors was a strategy in the pipeline for new anti-inflammatory compounds. These selective inhibitors, called coxibs, improved the efficacy of NSAIDs and diminished their damage to the gastrointestinal tract; however, they increased the risk of cardiotoxicity and hepatotoxicity [13]. For this reason, several drugs already approved by the Food and Drug Administration (FDA) were removed from the market. In addition to NSAIDs, glucocorticoids also represent the standard therapy for reducing inflammation. However, resistance to the anti-inflammatory effects of glucocorticoids constitutes a major drawback to the effective control of many diseases. Furthermore, glucocorticoid-associated side effects may involve metabolic disturbances, osteoporosis, and musculoskeletal, gastrointestinal, cardiovascular, neuropsychiatric, and immunological dysfunction [14,15]. Consequently, there is an urgent need to find novel, safe, and efficacious agents for the management of inflammation [1].

Accumulating data strongly suggest that phytochemicals from fruits, vegetables, nuts, and herbs may exert relevant beneficial effects due to their intrinsic antioxidant and anti-inflammatory properties [16]. The hermetic properties of phytochemicals were reported to activate adaptive stress response signaling pathways that increase cellular resistance to injury and disease. Thus, natural products as sources of new anti-inflammatory agents attracted increasing interest in the past decades. Among several naturally occurring bioactive substances, resveratrol (RSV, 3,4′,5-trihydroxystilbene), one of the most studied phytochemicals, entered the spotlight since the first scientific paper described its possible preventive effect on cancer in mice [17].

RSV is produced by plants as a phytoalexin in response to a stressful stimulus, or to a microbial or fungal infection, providing the plant resistance [18]. First isolated from *Veratrum grandiflorum* by Takaoka in the 1940s [19], RSV is found in food sources such as fruits, vegetables, and chocolate, and is better known as a constituent of grapes and wines, although it is present in only minimal quantities [18,20]. Due to its presence in wine, RSV attracted attention in the early 1990s to explain “the French paradox”, which suggested that people from France had a lower incidence of cardiovascular disease despite their high intake of saturated fats, presumably as a result of moderate red wine consumption [21].

The application of RSV attracted increasing interest not only from the pharmaceutical industries, but also from companies investing in cosmetics and food additives. Because of its potential as a topical anti-aging compound due to its downregulation of important transcription factors involved in photoaging, RSV gained popularity in dermatology applications as a cosmeceutical to improve skin health [22,23,24]. In addition, RSV is already widely distributed as an over-the-counter nutraceutical for its supposed beneficial effects on human health [20]. This increased interest in RSV activity resulted in a range of in vitro and animal studies demonstrating its beneficial effects. Several studies demonstrated the prophylactic and therapeutic properties of RSV in various diseases, including various types of cancer [25,26], diabetes [27], and cardiovascular diseases [28], which are linked by their important anti-inflammatory activity [20]. Furthermore, lifespan prolongation in several species was related to the desirable biological actions of RSV [29,30,31]. As a pharmacological tool, RSV has a broad spectrum of molecular targets, and it is believed that the observed effects result from its simultaneous action on multiple targets (summarized in Table 1). RSV generally modulates enzymes belonging to various classes, including kinases, lipoxygenases, cyclooxygenases, and sirtuins [32], and acts as a potent scavenger of free radicals [33].

Given the scientific interest in RSV over the past decade, this review focuses on understanding the anti-inflammatory effects of RSV against metabolic, cardiovascular, respiratory, and neurodegenerative diseases, as well as cancer.

## 2. Resveratrol Metabolism and Bioavailability

Mounting evidence indicates that RSV has a broad range of desirable biological actions. Despite its therapeutic properties, the application of these beneficial effects remains very limited. RSV exists as two geometric isomers: *cis* (Z) and *trans* (E). The *trans*-isomer is more abundant and biologically active than the *cis*-isomer. However, it was already demonstrated that RSV is extremely photosensitive, and 80–90% of the *trans*-RSV in solution is converted to *cis*-RSV upon exposure to light for 1 h [82]. Furthermore, the poor water solubility of RSV is another constraint for its biological application.

Although the oral absorption of RSV by humans is high (approximately 75%) [83,84], its bioavailability is less than 1% due to extensive intestinal and liver metabolism, involving glucuronic acid conjugation and sulfation that generate the key metabolites *trans*-resveratrol-3-O-glucuronide and *trans*-resveratrol-3-sulfate, respectively [83,85,86,87]. Since this polyphenol is known to have poor bioavailability in that it is rapidly metabolized and excreted, only trace concentrations of free RSV can be found in systemic circulation [83,85]. Therefore, the high concentrations of RSV commonly used for in vitro studies may not be physiologically relevant. Furthermore, the results of these studies are not expected to correlate well with those of in vivo studies, thus leading to disappointing outcomes in human clinical trials. Consequently, the successful clinical application of RSV is a severe challenge for the scientific community. To overcome these challenges, efforts were made to develop adequate drug delivery systems to achieve better clinical efficacy. These strategies include various approaches, such as the development of myriad RSV nanoformulations that can improve these inherent biologic limitations of RSV, increase its solubility, and prevent its degradation while preserving its biological activity [88,89,90,91,92].

## 3. Anti-Inflammatory Effects of Resveratrol on Metabolic Derangements and Cardiovascular Diseases

Metabolic disorders, such as obesity and type 2 diabetes, currently reach epidemic proportions, primarily due to a lifestyle based on calorie-rich diets and a lack of physical activity [93]. The incidence of obesity worldwide increased drastically during recent decades. A large part of this risk is due to obesity being a primary factor in the development of insulin resistance, type 2 diabetes, and metabolic syndrome, all of which create an increased risk of cardiovascular disease [94]. Furthermore, obesity is associated with an array of additional health problems, including increased risks of fatty liver disease; dyslipidemia, characterized by high plasma concentrations of triglyceride and low concentrations of high-density lipoprotein cholesterol; atherosclerosis; hypertension; degenerative disorders, including dementia; airway disease; and some cancers [94,95,96,97]. These metabolic derangements are all characterized by chronic low-grade inflammation, leading to the development of metabolic syndrome [94,95,96,97,98].

Some studies suggest that the effects of RSV on metabolic syndrome are associated with its ability to mimic caloric restriction, due to increased levels and activity of the protein deacetylase enzyme—silent information regulator 2/sirtuin-1 (SIRT1). SIRT1 plays a central role in the body’s response to diet and exercise [99,100]. In mice fed a high-calorie diet, several studies showed that long-term treatment with RSV improves factors associated with a longer lifespan, including increased insulin sensitivity [31,34,35,36], and reduced insulin-like growth factor-1 (IGF-1) levels [31]. RSV treatment also leads to increases in the metabolic rate and mitochondrial number, which might be correlated with increases in peroxisome proliferator-activated receptor-γ coactivator 1α (PGC-1α) activity and expression, which control mitochondrial biogenesis in the liver and muscle [31,34]. Additionally, weight loss [34,35,37], reduced fat mass [34], improvements in glucose homeostasis [34,37], and reductions in plasma triglyceride, tumor necrosis factor-alpha (TNF-α), and monocyte chemoattractant protein-1 (MCP-1) levels [37] were observed. In adipose tissues in mice, TNF-α, interferon (IFN)-β, IFN-α, and interleukin (IL)-6 levels were attenuated, as well as their upstream signaling molecules—toll-like receptors 2 and 4 (TLR2/4), myeloid differentiation primary response 88 (MyD88), and the transcription factor, nuclear factor kappa B (NF-κB) [37,38]. These findings were, in part, correlated with increases in AMP-activated protein kinase (AMPK) [31,34,36,38] and SIRT1 activity [35,38,101]. In addition, some clinical studies evaluated the effects of RSV in patients with metabolic syndrome, and achieved promising preliminary results, such as weight reduction [102], improved insulin sensitivity [103,104], and glycemic control [104,105]. However, further research should be conducted to confirm the pharmacological potential of RSV for treating the physiological changes of metabolic syndrome.

Although cardiovascular dysfunction might be linked to metabolic syndrome, cardiovascular disorders include any pathological condition of the blood vessels or heart leading to the obstruction of continuous blood supply and nutrients to cardiac tissue, and therefore, to the entire body [106]. Despite being largely preventable, cardiovascular diseases are the most common cause of death worldwide; they are responsible for almost one-third of all global deaths [107]. Furthermore, the number of worldwide deaths related to cardiovascular disorders is expected to reach 23.6 million in 2030 [28]. Inflammation was also established as a central driver of many disorders that affect the cardiovascular system [106]. Accumulating evidence shows that elevated circulating levels of inflammatory markers are associated with an increased risk of future cardiovascular events [108]. However, despite significant advances in cardiovascular research, much work remains to be done to reveal novel targets for therapeutic intervention [106]. Since RSV was linked to “the French Paradox” and shown to play a pivotal role in the protection of the cardiovascular system [28], accumulating evidence showed that its anti-inflammatory activity might underlie its protective mechanism against cardiovascular diseases.

Several in vitro studies revealed the anti-inflammatory effects of RSV in cardiac tissue, as evidenced by the inhibition of intercellular adhesion molecule 1 (ICAM-1), inducible nitric oxide synthase (iNOS), and IL-1β messenger RNA (mRNA) expression in human coronary arterial endothelial cells stimulated by TNF-α and treated with RSV [39]. Notably, it was already demonstrated that RSV inhibits TNF-α- and IL-6-induced increases in monocyte adhesion in primary human coronary arterial endothelial cells, which reduces pro-inflammatory NF-κB levels [40]. Another study showed that RSV decreases the level of eotaxin-1, a chemokine related to eosinophil recruitment, in human pulmonary artery endothelial cells stimulated with TNF-α or IL-13. This reduction was followed by the inhibition of the expression of the pro-inflammatory transcription factors, Janus kinase 1 (JAK1), phosphorylated extracellular signal-regulated kinase (ERK) 1/2, c-Jun N-terminal kinase (JNK), and signal transducer and activator of transcription (STAT) 6, and the reduction of the p65 subunit of NF-κB [109]. It is known that treatment with RSV also suppresses the bacterial lipopolysaccharide (LPS)-induced tissue factor expression in human peripheral blood mononuclear cells, which is the major initiator of the extrinsic blood coagulation pathway that is also involved with intracellular inflammation signaling [110]. Moreover, a study by Planavila and colleagues showed that RSV prevents phenylephrine, a hypertrophic agonist, or LPS-induced increases in MCP-1 levels in neonatal cardiomyocytes, suggesting that this effect is due to the activation of SIRT1 [41]. Csiszar et al. already showed the association between the anti-inflammatory effects of RSV and SIRT1 activation. Importantly, the authors showed that cultured coronary arterial endothelial cells stimulated with cigarette smoke extract, but previously treated with RSV, had decreased NF-κB transcriptional activity and iNOS, ICAM-1, IL-6, IL-1β, and TNF-α expression. Curiously, these effects were significantly attenuated by SIRT1 knockdown [42].

Cardiovascular diseases can result in heart failure, a progressive cardiac muscle disorder that leads to the deterioration of heart function, and results in the inability to meet the normal metabolic and energy needs of the body [111]. Some studies investigated the anti-inflammatory effects of RSV on heart failure using various animal models. A previous study showed that oral RSV treatment for 28 days significantly attenuated macrophage and mast-cell infiltration in the left ventricles of C57BL6 mice subjected to pressure overload-induced heart failure, induced by transverse aortic constriction surgery [112]. Furthermore, daily RSV intake for eight weeks resulted in cardioprotective effects against advanced-stage heart failure in rats subcutaneously injected with isoproterenol, a strong sympathetic agent used to induce myocardial infarction. Interestingly, this protective effect was accompanied by a reduction in pro-inflammatory members of the mitogen-activated protein kinase superfamily (p38-MAPK) and ERK1/2, suggesting that the regulation of these pro-inflammatory pathways may contribute to the beneficial effects of RSV in cardiac disorders [43].

Heart failure can also be attributable to the detrimental effects of acute myocardial ischemia/reperfusion injury [113], and anti-inflammatory effects of RSV on this type of injury were reported. In a recent study, Cong and co-workers showed that reduced myocardial infarction areas and myocardial myeloperoxidase levels, induced by RSV in a model of myocardial ischemia, were accompanied by decreased TNF-α concentrations in the serum and myocardium. Notably, these effects were abolished when the animals were treated with RSV combined with a nitric oxide (NO) synthase inhibitor and with a cyclic guanosine monophosphate (cGMP) inhibitor, indicating that these pathways are important for the anti-inflammatory activity of RSV [44]. Similarly, the authors showed that RSV also reduces the expression levels of NF-κB and TLR4, a known receptor that triggers innate immune responses; these findings further indicate the anti-inflammatory effects of RSV in protecting against myocardial ischemia [45]. These results are in line with previous work showing that RSV protects cardiomyocytes against anoxia/reoxygenation injury via the TLR4/NF-κB signaling pathway [46]. Hypertension is another factor that may drive the development of heart failure. RSV administration for eight weeks significantly reduced serum TNF-α and IL-6 levels in spontaneously hypertensive rats, but this treatment did not improve blood pressure [114]. These results suggest that combining RSV with blood pressure-lowering agents, which commonly do not affect the inflammatory profile, may provide optimal outcomes for reversing cardiovascular complications in hypertensive patients.

As discussed above, metabolic disorders, such as obesity and diabetes, increase the risk of cardiovascular disease development. Patients suffering from both diabetes and cardiovascular disease have a higher risk of mortality than patients without diabetes or heart failure [115]. Recently, a study showed that RSV improved cardiovascular functions in rats injected with streptozotocin, a compound toxic to pancreatic β cells. The improvement was linked to decreased serum levels of inflammatory factors, such as TNF-α, IL-1β, and IL-6, and the inhibition of vascular endothelial growth factor (VEGF), and the suppression of the p38-MAPK and NF-κB pathways [47]. Similarly, 12 weeks of RSV treatment reduced the circulating levels of TNF-α, IL-1β, and IL-6, and decreased the activation of the inflammatory factors angiotensin type 1 receptor (AT1R), ERK1/2, and p38-MAPK in rat hearts [48]. Furthermore, by treating mice with RSV for two months, Wu and co-workers found reduced serum, heart, and bone marrow-derived monocyte levels of high mobility group box 1 (HMGB-1), a pro-inflammatory cytokine that exerts its effects via binding to receptor for advanced glycation end products (RAGE) and toll-like receptors [116]. In line with these results, Delucchi and collaborators reported decreased HMGB-1 expression in left ventricular myocardial tissue in rats injected with streptozotocin and receiving a low dose of RSV [117].

Atherosclerosis is another coronary heart disease associated mainly with metabolic derangements, and the development of new therapies for this disorder is needed. This chronic disease is associated with arterial inflammation, lipid accumulation in the vessel wall, plaque formation, thrombosis, and late mortal complications, such as myocardial infarction and ischemic stroke [118]. Inflammatory responses play a crucial role in all phases of atherosclerotic development and progression, so the anti-inflammatory activity of RSV could be an interesting alternative for the control of the disease. In cultured THP-1-derived macrophages stimulated with LPS, pretreatment with RSV suppressed the formation of foam cells, which are considered to initiate atherosclerosis; in addition, the MCP-1 concentrations were reduced, and the expressions of SIRT1 and AMPK, a factor that is involved in glucose and lipid metabolism, and inhibits inflammation, were upregulated [49]. In a hyperlipidemia animal model in which rats were fed a cholesterol-enriched diet combined with vitamin D2, RSV treatment decreased the serum levels of IL-1β. Additionally, reduced MCP-1, ICAM-1, p65 NF-κB, and p38-MAPK mRNA and protein expression levels were found in the thoracic aortas of hypercholesterolemic rats treated with RSV, as well as decreased inflammasome nucleotide binding and domain-like receptor 3 (NLRP3) oligomerization. These effects were followed by the upregulation of SIRT1 mRNA and protein expression [50]. Interestingly, Chang and colleagues previously demonstrated that RSV reduces inflammatory markers, such as aortic macrophage infiltration and NF-κB expression, in an atherosclerosis model in which apolipoprotein E-deficient mice were fed a high-cholesterol diet [51]. Furthermore, an elegant study conducted by Cabo and co-workers showed that RSV prevented high fat and sucrose diet-induced arterial wall inflammation, and the accompanying increase in aortic pulse wave velocity in nonhuman primates [119]. Although more studies are warranted to understand the mechanisms involved in the anti-inflammatory effects of RSV on metabolic and cardiovascular disorders, the evidence discussed above can provide new options for the development of alternative therapeutic strategies using this polyphenol.

## 4. Respiratory Diseases and Resveratrol

Respiratory diseases are highly prevalent throughout the world, but occur mainly in westernized countries [120]. Despite therapeutic advances, there is a progressive increase in respiratory diseases, which seriously compromises human health [121]. The pathophysiological components and site of the inflammatory response may vary across respiratory diseases; however, the diseases share common characteristics, such as airway space oxidative stress increases, which play an important role in the lesion and inflammatory process [122]. RSV, as mentioned above, is widely known for its antioxidant and anti-inflammatory effects. Growing evidence indicates that RSV plays a protective role in respiratory diseases, which was already demonstrated in preclinical models of important respiratory conditions, such as chronic obstructive pulmonary disease (COPD), allergic inflammation (asthma models), and acute respiratory distress syndrome (ARDS).

COPD is a progressive lung disease with high global morbidity and mortality rates, and is characterized by persistent airflow obstruction and emphysema, which are caused primarily by cigarette smoke inhalation [123]. The mechanisms intrinsic to the pathophysiology of COPD are not yet fully elucidated, but the disease is associated with chronic inflammation that is usually resistant to corticosteroids [124]. In vitro assays using cells from a respiratory system treated with cigarette smoke extract, and cells from COPD patients demonstrated the anti-inflammatory and antioxidant effects of RSV. It was already shown that RSV reduces glutathione (GSH) depletion by augmenting GSH synthesis via activating nuclear factor (erythroid-derived 2)-like 2 (Nrf2), a redox-sensitive transcription factor [52]; RSV also inhibits COPD-related inflammatory mediators, such as IL-6, MCP-1, TNF-α, IL-8, and granulocyte-macrophage colony-stimulating factor (GM-CSF), and decreases nuclear NF-κB expression [53,125,126]. Cigarette smoke COPD models were used to show that RSV reduces lung histological damage, decreases pro-inflammatory protein levels (IL-6, IL-17, TNF-α, and transforming growth factor beta—TGF-β), inhibits airway remodeling, and reduces mucus hypersecretion [127]. RSV further alleviates the inflammation and reconstruction of small airways in the lungs by upregulating SIRT1 and PGC-1α expression [54]. In line with in vitro data, RSV treatment increases the activity of superoxide dismutase (SOD), GSH peroxidase, and catalase (CAT), as well as preventing the translocation of NF-κB to the nucleus and its binding activity [55].

Asthma is a heterogeneous clinical syndrome that mainly affects the lower respiratory tract; it is characterized by chronic inflammation, bronchoconstriction, increased airway hyperresponsiveness (AHR), and mucus production [128,129]. Current therapy consists of the combined use of short-acting β2 agonists and inhaled corticosteroids, as well as avoiding aggravating environmental factors [128]. In vivo studies over the past few years showed that RSV can effectively control asthma in murine models. RSV has anti-inflammatory effects by suppressing AHR [56,57,130,131], and reducing the infiltration of inflammatory cells, mainly eosinophils, into bronchoalveolar lavage fluid (BALF) [130] and lung tissue [56,57,58]. Total immunoglobulin E (IgE) and ovalbumin (OVA)-specific IgE levels were diminished in an OVA-induced asthma model, and reductions in IL-4, IL-5 [56,130], TNF-α [132,133], and TGF-β1 [57] cytokine levels were found. TGF-β1 and TGF-β1/phosphorylated Smad2/3 receptor expression levels in lung tissues were also significantly decreased with RSV treatment [57,131]. In addition to the anti-inflammatory effects, using RSV to treat asthma significantly downregulated oxidative stress by decreasing 8-isoprostane levels (an in vivo marker of oxidative stress) [56], reducing reactive oxygen species (ROS) production, and nicotinamide adenine dinucleotide phosphate (NADPH) oxidase cytosolic subunit p47phox expression, and enhancing SOD levels [133] and mitochondrial function [56]. Concerning airway remodeling, RSV attenuated the fibrotic response [132], and reduced sub-epithelial collagen deposition [131] and mucus hypersecretion [130]; RSV reduced mucus hypersecretion via inhibiting Mucin 5AC (Muc5AC), a major component of mucus [59]. The molecular mechanisms underlying the improvement of asthma include the increased lung expression levels of phosphatase and tensin homolog (PTEN) [58] and inositol polyphosphate 4 phosphatase (INPP4A), which are related to reduced protein kinase B (PKB/Akt) phosphorylation and activity [56]. It was also reported that RSV inhibits degranulation in mast cells and the protein expression of spleen tyrosine kinase (Syk), which plays an essential role in immune cell activation and lymphocyte development [132].

ARDS is an inflammatory disorder characterized by injury to the vascular endothelium and alveolar epithelium, pulmonary infiltration, and inflammatory mediator production. Various factors, such as pneumonia, sepsis, trauma, smoke, bacteria, and bacterial toxins, can lead to the development of ARDS [134,135]. Despite numerous efforts, there are currently no effective therapies for ARDS. A range of protocols to induce acute lung inflammation were used to demonstrate the beneficial activity of RSV in protecting against lung damage, and reducing inflammation through several possible molecular mechanisms. Similar data showed that RSV treatment improves structural changes in the lungs [60,136,137,138,139], decreases pulmonary edema [137,138,139], improves lung function [137], and diminishes neutrophil infiltration [134,137,138] and myeloperoxidase protein expression and activity in lung tissue [60,61]. Regarding cytokines, RSV significantly modulates IL-1β [60,139], IL-18 [60] IL-6, COX-2 [138], and macrophage inflammatory protein (MIP)-1α [139] in BALF and systemic TNF-α [61]. Its antioxidant effects are evidenced by reduced oxidative stress, including decreases in the pro-oxidant biomarker malondialdehyde (MDA) and hydrogen peroxide levels, increases in antioxidant biomarkers (GSH, CAT, and SOD activity) [136,140], and the inhibition of iNOS expression, ROS and NO production [60,139], and peroxynitrite formation [136]. These effects of RSV found in the ARDS model are associated with the downregulation of NLRP3 inflammasome activation through blocking NF-kB p65 nuclear translocation and its DNA-binding activity [60,138,139,141]. Moreover, the TLR4/Myd88 [138] and p38-MAPK [61,141] pathways are significantly downregulated by RSV.

## 5. Effects of Resveratrol on Neuroinflammation

Despite inflammation triggering complex molecular and cellular responses to neutralize and fight threats so as to restore normal body physiology, excessive or chronic inflammation damages the surrounding healthy tissues [142], and is considered to be actively involved in neurological disorders. For a long time, the brain was considered to be an immune-privileged organ [143]; however, the entire understanding of the interaction between the immune system and the central nervous system (CNS) was revised [144]. The immune system affects the CNS from its borders, and complex immune–CNS crosstalk was shown to play an essential role in protecting and supporting the CNS in health and disease [145,146]. Low-grade inflammation is linked to aging [147], and similar inflammatory processes are thought to occur in the periphery and in the brain, as evidenced by many studies reporting that the induction of systemic inflammation can trigger increased disease pathology in murine models of various CNS disorders [148]. In light of these new findings, immune–CNS interactions and neuroinflammation are recognized as common disease-escalating factors in many CNS pathologies [149]; these diseases include age-related dementia [150], and neurodegenerative diseases of the CNS, such as amyotrophic lateral sclerosis (ALS), Parkinson’s disease (PD), Alzheimer’s disease (AD), and multiple sclerosis (MS) [151,152,153,154].

The potential role of polyphenols in aging and neurodegeneration widened with discoveries that they modulate various important cell signaling pathways and sirtuins, a class of proteins involved in longevity and cell survival [29,30,31]. Given that the activation of inflammatory mechanisms is strongly associated with aging, and may underlie several CNS disorders, an expanding body of preclinical evidence suggests that RSV has the potential to impact a variety of CNS diseases. The hallmark of brain neuroinflammation is the reactive morphology of glial cells, including both astrocytes and microglia [155,156,157]. Upon activation, microglia secrete a range of pro-inflammatory factors, including prostaglandins, chemokines, cytokines, complement proteins, proteinases, ROS, and reactive nitrogen species, such as NO. The neuroprotective effects of RSV were described in several in vitro and in vivo models of CNS disorders.

RSV was found to reduce LPS-induced NO and TNF-α production in primary microglia [158], prevent LPS-induced microglial BV-2 cell activation [62], inhibit PGE2 and free radical production by rat primary microglia [159], and differentially modulate microglia and astrocyte inflammatory responses [160]. In addition, several studies used the N9 microglial cell line to indicate that RSV attenuated the LPS-induced phosphorylation of p38-MAPK and the degradation of inhibitor of κB (IκB), thus reducing the production of NO and TNF-α [158,161]. Furthermore, it was shown that RSV can prevent apoptosis in dopamine-producing neurons by inhibiting the production of microglia-derived TNF-α and IL-1β [162], and RSV can suppress *IL-6* gene expression and protein secretion in mixed glial cultures under hypoxia/hypoglycemia conditions [163].

Attenuating neuroinflammation is a therapeutic strategy for treating ischemic stroke, and several in vivo studies showed that RSV effectively reduces the increased expression of pro-inflammatory cytokines, inhibits NF-κB, reduces the phosphorylation of p38-MAPK and JNK activation via decreased COX-2 and iNOS expression, and inhibits astroglial and microglial activation induced by ischemia/reperfusion [164,165,166,167,168]. These findings suggest that the suppression of inflammation is associated with the neuroprotective effects of RSV, and RSV could be a promising candidate for stroke treatment.

Once microglia were shown to have functional plasticity and dual pro-inflammatory M1 and anti-inflammatory M2 phenotypes, Yang and collaborators reported that RSV suppressed microglia activation by promoting polarization toward the M2 phenotype via PGC-1α overexpression [63]. The increased M2 marker expression induced by RSV was accompanied by coactivation of the STAT6 and STAT3 pathways, and linked to the inhibition of NF-κB. The notion that RSV promotes PGC-1α expression could lead to the application of this polyphenol for PD therapy, as it was already demonstrated that PGC-1α expression and activation protect dopaminergic neurons in an MPTP mouse model of PD [64]. Interestingly, Jin and collaborators previously found that RSV decreased COX-2 and TNF-α levels in the substantia nigra of rats with 6-hydroxydopamine (6-OHDA)-induced PD [65]; however, thorough studies showing the mechanisms involved in the anti-inflammatory effects of RSV in PD are missing.

Microglial activation and inflammation were pointed out to play a pivotal role in AD pathogenesis. The basis for this assumption comes from studies showing that markers of inflammation such as TNF-α, IL-1β, IL-6, and other cytokines are increased in the brain, cerebrospinal fluid, and plasma of AD patients [10,169,170,171]. Mounting evidence suggests neuroinflammation induced by reactive microglia leads to reduced amyloid-β peptide (Aβ) clearance, triggers aberrant synaptic pruning [10,172,173], and prompts Aβ and tau pathologies. Taken together, these alterations contribute to impaired synapse function [174] resulting in memory dysfunction, the main characteristic of the disease. Although trials with anti-inflammatory compounds do not yet provide exciting outcomes [175], the lifelong use of NSAIDs was associated with a reduced risk of developing AD [176]. Therefore, polyphenols could provide new options for modulating neuroinflammation in AD.

Studies from our group demonstrated that RSV can protect organotypic hippocampal cultures from Aβ-induced toxicity through decreasing TNF-α, IL-1β, and IL-6 levels, and increasing IL-10 cytokine levels [66]. These salutary effects were highly correlated with the reduction of glial activation as a mechanism of protection. Corroborating data appear in a recent study showing that RSV inhibits neuroinflammation triggered by Aβ in cultured astrocytes and microglia [177]. Capiralla and colleagues previously showed that RSV mitigates LPS-induced NF-κB activation by interfering with TLR4 oligomerization, and IκB kinase (IKK) and IκB phosphorylation; these effects potently reduced the transcriptional stimulation of several NF-κB target genes, including TNF-α and IL-6, in RAW 264.7 macrophages and microglial BV-2 cells [62]. Furthermore, the authors showed that RSV prevented the pro-inflammatory effects of Aβ on macrophages by inhibiting IκB phosphorylation and activation of STAT1 and STAT3, and inhibiting TNF-α and IL-6 secretion. Interestingly, Wight and co-workers reported that RSV inhibited astrocyte production of NO, the cytokines TNF-α, IL-1β, and IL-6, and the chemokine MCP-1, which play critical roles in innate immunity. In addition, the authors also showed that RSV suppressed astrocyte production of IL-12p40 and IL-23, which are known to alter the phenotype of T cells that are involved in adaptive immunity [178].

TNF-α-dependent mechanisms appear to drive memory defects [179], thereby indicating a causal role of inflammation in the deleterious processes linked to AD. Our previous study suggests that the chronic administration of RSV blocked cognitive impairment in an animal model of AD, and this effect seemed to be related to the inhibition of synaptic dysfunction, and microglial and astroglial activation triggered by Aβ [67]. In addition, RSV treatment modulated important cell signaling pathways, such as the JNK, GSK-3β, and β-catenin pathways, which might be involved in neuroinflammation, cell metabolism, and survival. Importantly, the administration of RSV in a mouse model of cerebral amyloid deposition decreased the microglia activation associated with amyloid plaque formation [62,180]. Although a mechanistic link between inhibited microglia activation and the anti-inflammatory effects of RSV was not described in these studies, it is already known that microglial-derived cytokines enhance amyloid precursor protein (APP) processing, induce tau phosphorylation, and contribute to synapse plasticity impairment in neurons [174]. Altogether, these observations are consistent with the idea that RSV can modulate several signaling pathways involved in neuroinflammation.

## 6. Anti-Inflammatory Effects of Resveratrol on Cancer

Advances in diagnostic medicine during the past decades provided highly sensitive tools for early detection of cancer, one of the most commonly diagnosed diseases [181]. Development of cancer is a multistep process involving molecular and cellular alterations conventionally divided into initiation, promotion, and progression [182,183]. Together, these processes cause the hallmark of cancer: abnormal cell proliferation that cannot be controlled or stopped. Furthermore, epidemiological, pre-clinical, and clinical studies over the past several decades established a relationship between inflammation and cancer [184]. Inflammation is involved in all of the major steps of cancer initiation, metastasis progression, and drug resistance, and epidemiological studies suggest that as many as 25% of all cancers may be due to chronic inflammation [185,186]. Inflammatory signaling plays decisive roles in the development of cancer, and involves a complex interplay between oncogenic and tumor suppressive transcription factors [187].

Despite scientific breakthroughs during the past decades that expanded our knowledge regarding the cellular and molecular bases of cancer, the development of effective therapies with few side effects remains a challenge; therefore, the prevention of carcinogenesis is an area of considerable interest and research. Natural products may provide one the most promising approaches for reducing cancer via chemoprevention [188]. Given that inflammation is a critical component of tumor progression, and plays a key role in the tumor microenvironment, RSV might be a promising candidate for cancer prevention and/or treatment. RSV activity was documented in various cancer cell types; RSV acts on multiple targets and has anti-inflammatory effects, helping explain the plethora of anticancer pathways synergistically regulated by this polyphenol. After Jang and co-workers found that RSV inhibited carcinogenesis in a mouse skin cancer model in 1997 [17], a growing number of studies placed RSV at the center of anti-cancer research.

NF-κB regulates diverse cellular activities related to inflammation, and innate and adaptive immune responses [189], and the deregulation of NF-κB activity was implicated in the development of cancer [190,191]. Several in vitro, pre-clinical, and clinical studies showed that NF-κB and NF-κB-dependent gene expression play a major role in cancer progression, metastasis, and drug resistance [192]. The anti-inflammatory effects of RSV seem to be related to NF-κB signaling attenuation [193,194], which drives the inhibition of the growth and invasion of various tumor types [22,68,69,70,71,195]. In colorectal cancer cells, for instance, RSV attenuates the phosphorylation, acetylation, and nuclear translocation of NF-κB [72]. Furthermore, RSV was reported to inhibit iNOS expression in colon cancer cells [73]; iNOS is an enzyme induced by cytokines and pro-inflammatory agents. The overproduction of NO can result in cellular injury and inflammation, and plays an important role in colon tumorigenesis. Because the expression and activity of iNOS were demonstrated in human colorectal cancer tissue and animal models [196], the use of RSV as a chemopreventive agent should be investigated. Additionally, it was shown that RSV inhibits the IGF-1R/Akt/Wnt pathways, and activates p53 [74] to impair cell development and tumorigenesis. In line with these findings, Frazzi and co-workers showed that RSV, through modulation of SIRT1, induces increased p53 expression and acetylation, leading to an increase in apoptosis and cell death of a Hodgkin-lymphoma cell line [197]. Therefore, RSV can constitute a promising agent affecting cancer initiation.

Alterations in the Phosphatidylinositol-4,5-bisphosphate 3-kinase (PI3K)/Akt signaling pathway are frequent in human cancer. PI3K/Akt signaling is associated with cell proliferation and survival, and plays a major role in tumor growth, as well as the potential response of a tumor to cancer treatment [198]. Further, PI3Kγ regulates the chemokine-mediated recruitment and activation of immune cells [199], and Akt regulates the transcriptional activity of NF-κB by inducing the phosphorylation and subsequent degradation of IκB [200]. Mounting evidence shows that RSV, paradoxically, can inhibit the PI3K/Akt pathway in cancer to regulate cell differentiation, growth, proliferation, and several other activities [75,76,201]. Additionally, it was reported that RSV inhibits the adhesion, invasion, and migration of glioblastoma-initiating cells, both in vitro and in vivo, through suppressing the PI3K/Akt/NF-κB cascade [77]. Given that TNF-α is a strong stimulatory factor in various cancer cell lines [202], previous studies reported that RSV inhibits TNF-α-induced cell invasion in many types of cancer cells through inhibiting NF-κB [68,195]. Furthermore, RSV represses the expression of IL-6, B-cell lymphoma 2 (BCL-2), BCL-xL, X-linked inhibitor of apoptosis protein, cellular inhibitor of apoptosis protein, VEGF, and matrix metalloproteinase-9 (MMP-9), the syntheses of which are regulated by NF-κB [78,203]. In accordance with these studies, Ryu and colleagues reported that RSV reduces TNF-α-induced U373MG human glioma cell invasion through regulating NF-κB activation [69].

The maintenance of a pro-carcinogenic inflammatory microenvironment linked to multiple alterations in cell signaling pathways was recognized to play a key role in the transitions from a normal cell to a neoplastic malignant cell, as well as during cancer progression. Although our current understanding of these alterations is limited, STAT3 seems to be a critical element in inflammation-related tumorigenesis fostering the proliferation, survival, invasion, and angiogenesis of tumor cells [204]. Additionally, the signaling mediated by STAT3 is interconnected with NF-κB signaling, and the co-regulation of inflammatory and oncogenic genes, such as those coding for IL-1β, BCL-xL, Myc, COX-2, and cyclin D1 [204]. Importantly, this interaction can further promote the development of tumors via inducing the expression of pro-tumorigenic genes [79]. It is noteworthy that RSV inhibits proliferation, induces apoptosis, and overcomes chemoresistance through downregulation of STAT3 and NF-κB [79], providing support for its pro-apoptotic and anti-proliferative potential. In line with these observations, it was recently found that RSV could modulate the mitogen-activated protein kinase (MAPK) pathway. Despite needing a constitutively active state of MAPKs for the maintenance of the malignant state, short-term MAPK activation may cause cell apoptosis [205]. At low concentrations, RSV activates ERK1/2; however, at higher concentrations, it can inhibit MAPK [80]. In cervical carcinoma cells, RSV inhibits the activation of p38, JNK1, and ERK2 [81]. In contrast, RSV activates ERK1/2 in prostate [206], breast [207,208], glial [209], and ovarian cancer cells [210].

Another outstanding effect of RSV against cancer promotion and progression is related to the control of the expression of microRNAs (miRNAs), mainly those at the crossroads of inflammation, cell differentiation, and homeostasis. For instance, RSV activity appears to be partially dependent on the impaired expression of miR-663, miR-21, and miR-155, which are linked to tumor suppression, oncogenicity, and pro-inflammatory effects, respectively. Modulation of these miRNAs by RSV led to decreased secretion of pro-inflammatory cytokines IL-6, IL-8, and TNF-α, reduced expression of adhesion proteins, such as ICAM-1, and leukocyte chemoattractants, and increased production of anti-inflammatory cytokines [211]. Overall, these observations indicate the anti-inflammatory effects of RSV on various types of cancer, and provide new directions for RSV chemoprevention, and its use as a chemotherapeutic agent.

## 7. Conclusions

**Figure 1:**
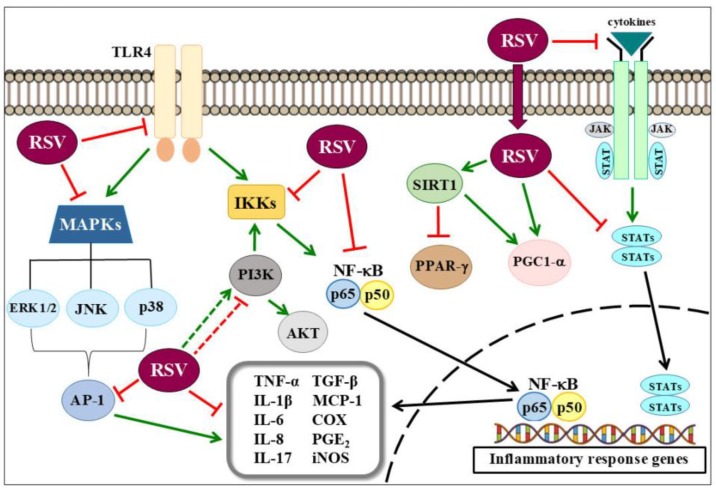
Some of the molecular bases of resveratrol anti-inflammatory effects. Inflammation induces the activation of several cell signaling pathways. The exact mechanism of RSV-mediated protection is not yet understood, but it was described that RSV interacts with multiple targets, and alters dysregulated inflammatory pathways and mediators. Arrows with a point indicate activation, while arrows with a flat tip indicate inhibition. Dashed arrows indicate a poorly understood mechanism.

Extensive research within the past several decades revealed that chronic, low-grade inflammation can underlie the development of several non-communicable diseases, including cancer, and neurodegenerative, respiratory, metabolic, and cardiovascular diseases. Although scientific breakthroughs during the past decades expanded our knowledge of the cellular and molecular mechanisms underlying inflammation, this knowledge is not yet translated into effective therapies. Accumulating data strongly suggest that phytochemicals can interact with multiple targets, and alter the dysregulated inflammatory pathways and mediators, thus indicating the possible development of affordable, novel, and safe drugs for the treatment of inflammatory processes underlying chronic diseases. The growing interest in RSV increased our understanding of how this polyphenol can directly and indirectly modulate major signaling pathways that protect cells from inflammation (Figure 1). While preclinical studies yielded exciting results, there is little clinical evidence that RSV is an effective therapeutic in humans. Although some official systematic clinical trials using RSV treatment in humans had some disappointing outcomes, the difficulties of the clinical application of RSV are enormous, such as its poor water solubility, bioavailability, and dosage. Therefore, in-depth scientific investigations and large-scale clinical trials are required to completely determine the potential of RSV, and provide new options for the better management of inflammation in patients with chronic diseases.

## Figures and Tables

**Table 1:** Anti-inflammatory effect of Resveratrol in chronic diseases.

Disease	Model	Concentration/Dose	Inflammatory Molecules Affected	Ref.
Cardiovascular and Metabolic Disorders	High-fat diet in AMPK knockout mice model	400 mg/kg v.o.	AMPK	[34]
	High-fat diet mice model	30–400 mg/kg v.o.	↑ PGC-1α expression ↑ SIRT1 activation ↑ AMPK phosphorylation ↓ TLR2/4, MyD88, NF-κB and AMPKα expression	[35,36,37,38]
	TNF-α-stimulated human coronary arterial endothelial cells	1–100 µM	↓ ICAM-1 and iNOS expression ↓ NF-κB activation	[39,40]
	Phenylephrine or LPS-stimulated neonatal cardiomyocytes	50 µM	SIRT1-dependent	[41]
	Cigarette smoke extract-stimulated rat arteries and cultured coronary arterial endothelial cells or Cigarette smoke-exposed rats	10 µmol/L or 25 mg/Kg in drinking water	↓ iNOS, ICAM-1 and NF-κB expression ↑ SIRT1	[42]
	Postinfarction heart failure murine model	15 mg/Kg in drinking water	↓ p38-MAPK and ERK1/2 expression	[43]
	Ischemia/reperfusion murine model	100 µmol/L, i.v.	↓ NO and GMPc-dependent ↓ NF-κB and TLR4 expression	[44,45]
	Cardiomyocytes anoxia/reoxygenation injury in vitro model	5-20 µM	↓ NF-κB and TLR4 expression	[46]
	Cardiovascular disorder in streptozotocin-induced diabetic rats model	0.75–80 mg/Kg i.g.	↓ NF-κB level ↓ VEGF expression ↓ p-p38 expression ↓ ERK1/2 and AT1R expression	[47,48]
	LPS-stimulated THP-1-derived macrophages	2.5 µM	↑ SIRT1 and AMPK expression	[49]
	Atherosclerosis model induced by hypercholesterolemia in rats	50 mg/kg in daily diet	↓ ICAM-1, NF-κB and p38-MAPK expression ↑ SIRT1	[50]
	Atherosclerosis model induced by hypercholesterolemia in (apo E)-deficient mice	25 mg/Kg, v.o.	↓ NF-κB expression	[51]
Respiratory Diseases	Cigarette smoke stimulated human lung epithelial cells	10 µM	↑ Nrf2 expression	[52]
	Non-stimulated human lymphocyte	12.5 µmol/L	↓ NF-κB expression	[53]
	Cigarette smoke exposure + LPS rats model	50 mg/kg v.o.	↑ SIRT1 and PGC-1α expression	[54]
	Cigarette smoke exposure mice model	1–3 mg/ kg v.o.	↓ NF-κB nuclear translocation	[55]
	OVA-induced mice asthma model	10–50 mg/kg v.o.	↑ NPP4A expression ↓ Akt phosphorylation ↓ TGF-β1/phosphorylated Smad2/3	[56,57]
	OVA-induced mice asthma model	30 mg/kg i.p.	↑ PTEN expression ↓ MUC5AC expression	[58,59]
	LPS-induced mice ARDS model	5–30 mg/kg i.p.	↓ NF-κB p65 nuclear translocation ↓ p38 MAPK expression	[60,61]
Neuroinflammation	LPS-induced murine RAW 264.7 macrophages and microglial BV-2 cells	25–100 µM	↓ TLR4 oligomerization ↓ NF-κB activation ↓ IκB kinase and IκB phosphorylation ↓ STAT1/3 signaling	[62]
	LPS- stimulated mouse microglia BV2 cells	5–50 µM	↑ PGC-1α expression ↓ NF-κB p65 translocation	[63]
	Neurotoxin MPTP- stimulated dopaminergic SN4741 cells	5–10 µM	↑ PGC-1α expression	[64]
	6-OHDA induced Parkinson’s rat model	20 mg/kg v.o.	↓ COX-2	[65]
	AD model induced by Aβ	5–10 µM5 mg/kg i.p.	↓ GFAP ↓ JNK and GSK-3β activation ↓ p-β-catenin	[66,67]
Cancer	TNF-α-stimulated HepG2 human hepatocellular carcinoma cells	10–100 µM	↓ NF-κB expression	[68]
	TNF-α-stimulated U373MG human glioma cell	5–20 µM	↓ NF-κB and uPA and uPAR expression	[69]
	Helicobacter pylori-induced gastric inflammation in mice	100 mg/kg, v.o.	↓ IκBα phosphorylation and iNOS expression ↑ Nrf2 expression	[70]
	3D aggregates of SKOV-3 and OVCAR-5 ovarian cancer cell	10–30 µM	↓ NF-κB expression	[71]
	HEK293T human embryonic kidney cells transfected with NF-B Luc vector	10–40 µg/mL	↓ NF-κB activity and IKK-mediated NF-κB activation	[72]
	LPS-stimulated Caco-2 and SW480 human colon cancer cells	10–50 µM	↓ IκBα phosphorylation ↓ iNOS expression and TLR4 expression	[73]
	HT-29 and SW480 human colon cancer cell lines	100–150 µM	↓ IGF-1R/Akt and Wnt/β-catenin signaling pathway ↑ p53 protein	[74]
	Human bladder cancer cell line T24 or xenograft cancer model in mice	50–200 µM or 20 mg/Kg, i.p.	↓ Akt expression	[75]
	HepG2 Human hepatocellular carcinoma and Chang liver cells	200 µM	↓ p38 MAP kinase and PI3K/Akt expression	[76]
	Glioblastoma-initiating cells or xenograft cancer model in mice	5–20 µM or 10 mg/Kg, i.p.	↓ PI3K/Akt and NF-κB expression	[77]
	RPMI 8226, U266, and KM3 multiple myeloma cell lines	100–200 µM	↓ NF-κB expression	[78]
	U266 and RPMI 8226 multiple myeloma cells	50 µM	↓ NF-κB expression ↓ STAT3 activation	[79]
	SH-SY5Y human neuroblastoma cells	50–100 µM	↓ ERK1/2 phosphorylation	[80]
	HeLa human cervical squamous carcinoma cells	50 µM	↓ JNK, p38, and ERK2 activities	[81]

v.o. = via oral; i.v. = intravenous; i.g. = intragastric; i.p. = intraperitoneal; ↑= enhances and ↓= decreases.

## Abbreviations

6-OHDA	6-Hydroxydopamine
Aβ	Amyloid-Beta Peptide
AD	Alzheimer’s Disease
AHR	Airway Hyperresponsiveness
ALS	Amyotrophic Lateral Sclerosis
AMPK	AMP-Activated Protein Kinase
ARDS	Acute Respiratory Distress Syndrome
AT1R	Angiotensin Type 1 Receptor
BALF	Bronchoalveolar Lavage Fluid
BCL-2	B-Cell Lymphoma 2
CAT	Catalase
cGMP	Cyclic Guanosine Monophosphate
CNS	Central Nervous System
COPD	Chronic Obstructive Pulmonary Disease
COX	Cyclooxygenases
ERK	Extracellular Signal-Regulated Kinase
GM-CSF	Granulocyte-Macrophage Colony-Stimulating Factor
GSH	Glutathione
HMGB-1	High Mobility Group 1
ICAM-1	Intracellular Adhesion Molecule 1
IFN	Interferon
IGF-1	Insulin-Like Growth Factor-1
IKK	IκB Kinase
IκB	Inhibitor of Kappa B
IL	Interleukin
iNOS	Inducible Nitric Oxide Synthase
INPP4A	Inositol Polyphosphate 4 Phosphatase
JAK	Factors Janus Kinase 1
JNK	C-Jun N-Terminal Kinase
LPS	Lipopolysaccharides
MAPK	Mitogen-Activated Protein Kinase
MCP-1	Monocyte Chemoattractant Protein-1
MDA	Malondialdehyde
MMP-9	Matrix Metalloproteinase-9
MPTP	1-Methyl-4-Phenyl-1,2,3,6-Tetrahydropyridine
MS	Multiple Sclerosis
NLRP3	Nucleotide Binding and Oligomerization Domain-Like Receptor 3
NO	Nitric Oxide
NSAIDs	Non-Steroidal Anti-Inflammatory Drugs
OVA	Ovalbumin
PD	Parkinson’s Disease
PG	Prostaglandins
PGC-1α	Peroxisome Proliferator-Activated Receptor-γ Coactivator 1α
PTEN	Phosphatase and Tensin Homology Deleted on Chromosome Ten Gene
RAGE	Advanced Glycation End Products
ROS	Reactive Oxygen Species
RSV	Resveratrol
SIRT-1	Sirtuin 1
SOD	Superoxide Dismutase
STAT	Signal Transducer and Activator of Transcription
TGF-β	Transforming Growth Factor Beta
TLR4	Toll-Like Receptor 4
TNF-α	Tumor Necrosis Factor-Ahlpa
uPA	Urokinase Plasminogen Activator
uPAR	Urokinase Plasminogen Activator Receptor
VEGF	Vascular Endothelial Growth Factor
